# The vaccinia virus K7 protein promotes histone methylation associated with heterochromatin formation

**DOI:** 10.1371/journal.pone.0173056

**Published:** 2017-03-03

**Authors:** Wondimagegnehu M. Teferi, Megan A. Desaulniers, Ryan S. Noyce, Mira Shenouda, Brittany Umer, David H. Evans

**Affiliations:** Department of Medical Microbiology & Immunology, Li Ka Shing Institute of Virology, University of Alberta, Edmonton, Alberta, Canada; Saint Jude Children's Research Hospital, UNITED STATES

## Abstract

It has been well established that many vaccinia virus proteins suppress host antiviral pathways by targeting the transcription of antiviral proteins, thus evading the host innate immune system. However, whether viral proteins have an effect on the host’s overall cellular transcription is less understood. In this study we investigated the regulation of heterochromatin during vaccinia virus infection. Heterochromatin is a highly condensed form of chromatin that is less transcriptionally active and characterized by methylation of histone proteins. We examined the change in methylation of two histone proteins, H3 and H4, which are major markers of heterochromatin, during the course of viral infection. Using immunofluorescence microscopy and flow cytometry we were able to track the overall change in the methylated levels of H3K9 and H4K20. Our results suggest that there is significant increase in methylation of H3K9 and H4K20 during *Orthopoxviruses* infection compared to mock-infected cells. However, this effect was not seen when we infected cells with *Leporipoxviruses*. We further screened several vaccinia virus single and multi-gene deletion mutant and identified the vaccinia virus gene K7R as a contributor to the increase in cellular histone methylation during infection.

## Introduction

Poxviruses employ large DNA genomes and replicate in the cytoplasm of infected cells. Nevertheless, a significant number of poxvirus gene products can be detected in the nucleus of cells for reasons that are more or less well understood. These include the vaccinia virus (VACV) C4 [1], C6 [2], C16 [3], B14 [4], E3 [5], F16 [6], and N2 [7] gene products as well as the myxoma virus M148 [8] and M150 [9] proteins. Many of these proteins target systems that regulate innate immune defenses against viruses, for example C4 and B14 inhibit NF-κB activation, while C6 and N2 inhibit IRF3 activation. A common feature of these systems is that the virus protein binds to one or more of the cellular proteins that play a role in regulating gene transcription, and thus prevents activating the associated antiviral pathway(s).

Whether any of these virus-encoded proteins can exert a global effect on host transcription is less well established. A number of microarray and RNA sequencing studies have examined the changes in the cellular transcriptome during Orthopoxvirus infection [10–12]. Many of these studies have concerned VACV, where one observes a general decrease in expression of most cellular genes in virus-infected cells, especially at late time points in infection [11, 12]. This can be attributed in part to cellular transcripts being destabilized by the activity of two virus-encoded mRNA decapping enzymes D9 and D10 [13]. However, not all cell transcripts behave in this manner, there are a few classes of genes that were up regulated throughout infection, including some types of genes involved in intracellular signal transduction, while a few other genes show unchanged transcription profiles [10–12].

Of course nuclear gene expression can be regulated in other ways. In particular, a great many epigenetic regulatory mechanisms have been discovered that also affect gene activity. Many of these systems act by modulating the structure of the histone-DNA complexes, or nucleosomes, that comprise chromatin. Chromatin varies greatly in the degree of compaction across different chromatin domains or states [14] and is often described as being active or repressed [15]. Euchromatin is less condensed and usually transcriptionally active, whereas heterochromatin is highly condensed and transcriptionally less active. Heterochromatin can be further categorized into constitutive and facultative heterochromatin (reviewed in [16]). Constitutive heterochromatin is found in stably condensed regions of the nucleus whereas facultative heterochromatin can be found in euchromatic regions and varies in extent depending upon the cell environment.

Chromatin structure is commonly modulated through post-translational modification of core histone N-terminal tails. Many types of histone modifications are known [17], but histone methylation is of most relevance to this communication and plays a critical role in the regulation of transcription, DNA replication, DNA repair, cell cycle, nuclear architecture, embryogenesis and development (reviewed in [18]). Heterochromatin is generally marked or tagged by methylated lysines in the N-termini of histones H3 and H4 (H3K9me2, H3K9me3, and H4K20me3 [15, 18]). While H3K9me2 and H3K9me3 are predominantly associated with the repetitive sequences found in constitutive heterochromatin, they are also found in broad chromatin domains within euchromatic regions. Moreover, H3K9me2 and H3K9me3 are found in facultative heterochromatin at promoters and gene bodies subject to transcriptional suppression. H3K9me3 marks are also deposited in areas containing double-strand DNA breaks, where they probably cause transient suppression of gene expression until repair is completed [19]. These methylation reactions are catalyzed by a family of methyltransferases [20]. For example, the H3K9 marks in constitutive heterochromatin are predominantly added by SUV39H1 and SUV39H2 [21, 22], although these enzymes also play a role in producing facultative heterochromatin through H3K9 tri-methylation at promoters and at double-strand DNA breaks [19].

Our interest in studying the interaction between poxviruses and the cellular chromatin originated from a project that explored the biology of VACV-encoded DNA-binding proteins. One of the predicted VACV genes encodes the 40 kDa E5 protein bearing two putative DNA-binding BEN domains [23] spanning residues 102–212 and 223–318 (prosite.expasy.org). BEN domains are found in a variety of animal and virus proteins, and are thought to play a role in chromatin regulation and transcriptional suppression [24, 25]. Thinking that E5 might play an unknown role in modulating chromatin structure, we examined how VACV infection affected the levels of repressive chromatin found in the presence and absence of the E5R gene. Interestingly, both wild type and mutant (ΔE5R) VACV infections caused a similar increase in the levels of markers of repressive or facultative chromatin. Given that E5 is currently thought to be a predominantly cytoplasmic virosome-associated protein [26], this result may not be too surprising, however it leaves unanswered the question of what VACV gene(s) might be causing these changes associated with alterations in chromatin structure. In this study we analyzed how cellular chromatin tags change during poxvirus infection with a specific focus on the H3K9 and H4K20 methylation that leads to formation of repressive chromatin. Our analysis shows that Orthopoxviruses, but not Leporipoxviruses, promote formation of H3K9me3 and H4K20me3 histone tags and that these reactions are dependent (in part) on the activity of the VACV K7R gene product.

## Results

### VACV infection increases the levels of markers of repressive chromatin

**Fig 1:**
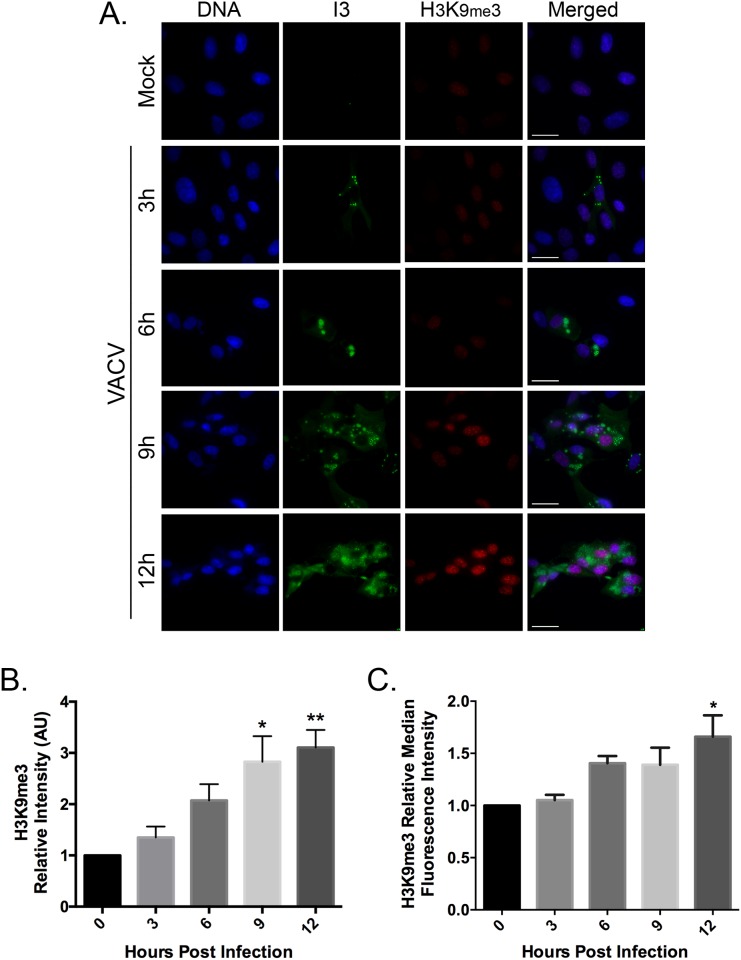
VACV infection increases the levels of H3K9me3. (**A)** BSC-40 cells were grown on coverslips and infected at a MOI 5.0 with VACV strain WR. At various time points post infection, the coverslips were fixed and stained to detect the VACV I3 protein and H3K9me3. DNA was counterstained with DAPI. Images were acquired using an Olympus IX-71 inverted microscope at 60x magnification and deconvolved using Softworx software (GE Healthcare) (scale bar = 25 μm). The nuclear H3K9me3 signal intensities from (**B**) microscopy images and (**C**) flow cytometry were quantified using FIJI imaging analysis software and normalized to mock-infected cells. At least five images were analyzed per samples within an independent experiment. Data represent the standard error of the mean (SEM) of three independent experiments. GraphPad was used to determine significant differences in H3K9me3 levels following VACV infection. Statistically significant differences are noted, relative to time zero (*P<0.05; **P<0.01).

Repressive chromatin is characterized by a compact structure and histones enriched with H3K9me3, H4K20me3 and H3K27me3 [27, 28]. These markers are found in both constitutive and facultative heterochromatin domains. To examine the effects of VACV infection on the amounts and distribution of H3K9me3, BSC-40 cells were infected with VACV strain Western Reserve (WR) at a multiplicity of infection (MOI) of five and analyzed by immunofluorescence microscopy and flow cytometry. We saw a global increase in the intensity of nuclear H3K9me3 staining in infected cells as well as an increase in the number of nuclear H3K9me3 foci (Fig 1A). When cells were harvested, fixed, and stained at different time points following infection, these levels increased approximately linearly throughout the first 12 hr of the experiment. By 9–12 hr post-infection, levels of H3K9me3 showed a significant increase of ~3-fold relative to mock infected cells (Fig 1B). The increase in H3K9me3 staining was seen primarily in infected cells, judging by the co-staining for the virus infection marker I3. I3L encodes the VACV single-strand DNA binding protein [29] and I3 is expressed at high levels throughout the infection cycle. Flow cytometry was used to corroborate the results of the experiments performed using immunofluorescence microscopy (Fig 1C). By 12 hr post-infection the H3K9me3 signal was elevated about 1.5-fold relative to uninfected cells. Collectively both methods show that VACV infection significantly increases H3K9me3 levels.

**Fig 2:**
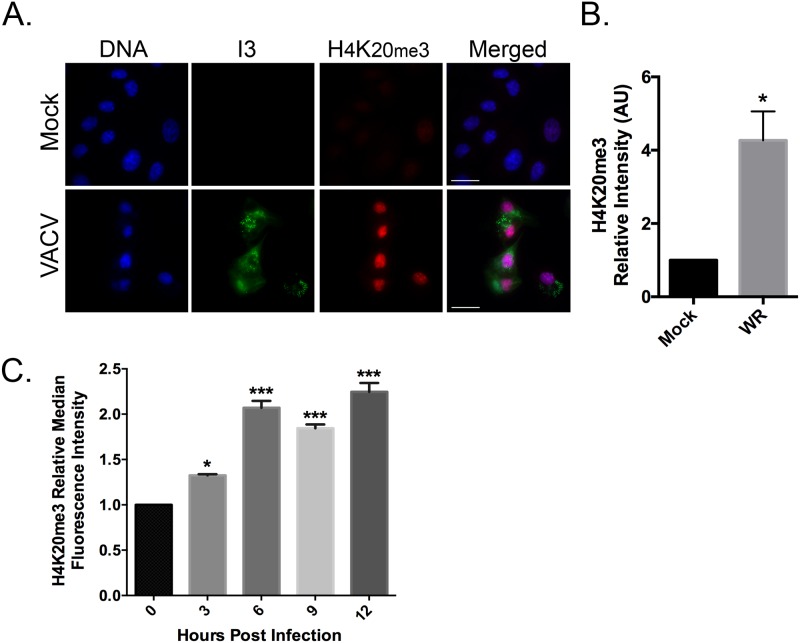
VACV infection increases the levels of H4K20me3. (**A)** BSC-40 cells were grown on coverslips and infected at a MOI 5.0 with VACV. The cells were fixed and stained to detect VACV I3 protein, H4K20me3, and DNA 9 hr post-infection. Images were acquired at 60x magnification (scale bar = 25 μm). The nuclear H3K9me3 signal intensities from (**B**) microscopy images and (**C**) flow cytometry were quantified using FIJI imaging analysis software and normalized to mock-infected cells. At least five images were analyzed per samples within an independent experiment. The experiment was performed three independent times and the SEM was then calculated relative to the mock infection **(B)** or time zero **(C)**. Statistically significant differences are noted (*P<0.05; ***P<0.001).

Repressive chromatin is also enriched in H4K20me3, which is produced by SUV39H1/2 acting downstream of H3K9 tri-methylation during heterochromatin formation [28, 30]. To determine if VACV infection also alters the levels of this marker for repressive chromatin, H4K20me3 levels were monitored by immunofluorescence microscopy and flow cytometry. As with H3K9me3, VACV infection also caused a global increase in the intensity and distribution of H4K20me3 foci. Quantification of the nuclear H4K20me3 levels detected a ~ 4 fold increase in H4K20me3 9h post-infection, relative to uninfected cells (Fig 2A and 2B). Flow cytometry was also used to verify the results observed by immunofluorescence. Infected cells that expressed H4K20me3 significantly increased ~2 fold throughout the infection (Fig 2C). Collectively these results show that VACV infection increases the levels and distribution of markers of repressive chromatin, with maximum effect seen 9–12 hr post-infection in BSC-40 cells.

### Not all poxvirus infections promote H3K9me3 and H4K20me3 formation

**Fig 3:**
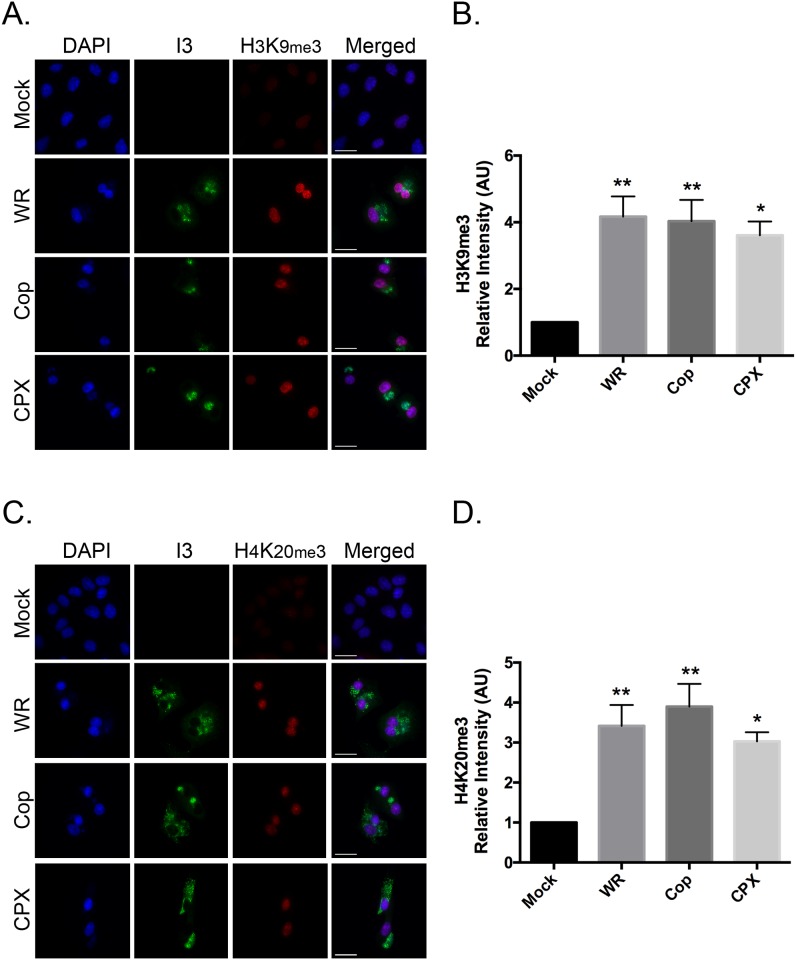
Orthopoxviruses promote H3K9me3 and H4K20me3 formation. BSC-40 cells were grown on coverslips and subsequently infected with VACV WR, VACV Cop, and CPXV. The cells were fixed and stained to detect I3 and (**A**) H3K9me3 or (**C**) H4K20me3 9hr post-infection. DNA was counterstained with DAPI. Images were acquired at 60x magnification (scale bar = 25 μm). The nuclear (**B**) H3K9me3 and (**D**) H4K20me3 signal intensities were quantified using FIJI imaging analysis software and normalized to mock-infected cells. Data represent the SEM of three independent experiments and any statistically significant differences relative to mock-infected cells, are noted (*P<0.05; **P<0.01).

To determine whether the changes in the levels of markers of repressive chromatin was a feature characteristic of *Orthopoxvirus* infections in general; the immunofluorescence microscopy was repeated using other poxviruses. The cells were infected with different viruses at MOI = 5, fixed 9h post-infection, and the levels of H3K9me3 (Fig 3A and 3B) and H4K20me3 (Fig 3C and 3D) were determined by fluorescence microscopy. Both VACV (strain Copenhagen; Cop) and cowpox virus (CPX) increased the levels of H3K9me3 and H4K20me3 marks to an extent similar to what was seen in cells infected with VACV strain WR. That is the levels of H3K9me3 (Fig 3B) and H4K20me3 (Fig 3D) increased 3- to 4-fold in VACV Cop and CPX-infected cells, relative to uninfected cells.

**Fig 4:**
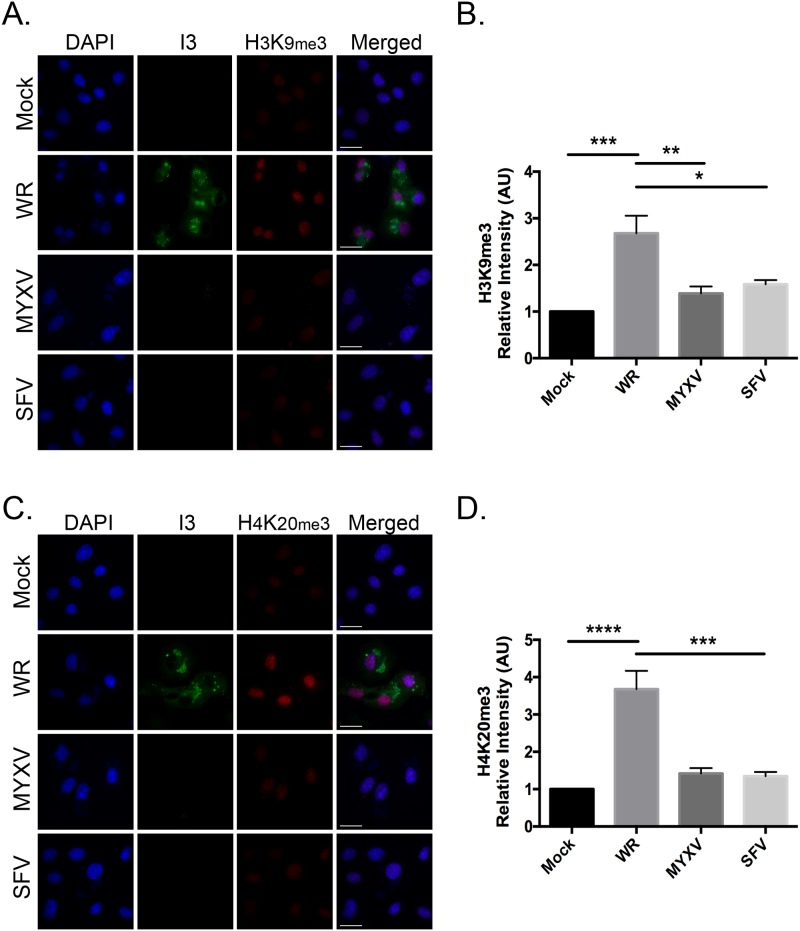
Leporipoxvirus infection does not increase the levels of H3K9me3 and H4K20me3 formation. BSC-40 cells were grown on coverslips and subsequently infected with VACV WR, MYXV, or SFV. Eighteen hours post infection the cells were fixed and stained to detect VACV I3 and (**A**) H3K9me3 or (**C**) H4K20me3. DNA was counterstained with DAPI. The presence of viral factories (stained with DAPI) was used to confirm infection with MYXV and SFV, as the I3 antibody does not cross-react in Leporipoxviruses. Representative images are shown (scale bar = 25 μm). Nuclear (**B**) H3K9me3 and (**D**) H4K20me3 signal intensities were quantified using FIJI imaging analysis software and normalized to mock-infected cells. We show the SEM of three independent experiments. Statistically significant differences are noted (*P<0.05; **P<0.01; ***P<0.001; **** *P*<0.0001).

In contrast to what was seen in cells infected with VACV and cowpox virus, myxoma virus (MYXV) and Shope fibroma virus (SFV) did not significantly alter the levels of H3K9me3 (Fig 4A and 4B) and H4K20me3 (Fig 4C and 4D) when these were measured optically at 9 hr post infection. Because Leporipoxviruses replicate more slowly than Orthopoxviruses, we extended the time course. However, no increase in levels of H3K9me3 beyond the background, were detected as late as 18 hr post-infection either. Thus, the effects we see are characteristic of *Orthopoxviruses* and may not be generalizable to all genera of poxviruses.

To confirm that the increase in H3K9me3 marks on host heterochromatin was not limited only to BSC-40 cells, we also infected primary human embryonic lung (HEL) fibroblasts with VACV or SFV (S1 Fig). The level of H3K9me3 marks was increased following VACV infection, although in these cells the nuclei also acquired a crescent shape, with nuclear blebbing sometimes being observed. In contrast, we did not see any significant changes in the levels of H3K9me3 in SFV-infected HEL cells (S1 Fig), nor did we see any change in the morphology of the nuclei in SFV-infected cells even though early factories could be seen showing that SFV can establish infection in these cells. The change in nuclear morphology at an early time post infection complicates the interpretation of these data, as do the differences in host range of VACV *versus* SFV. However, these observations do show that VACV infection in primary human fibroblasts can still promote the formation of H3K9me3 marks characteristic of repressive chromatin.

### H3K9me3 and H4K20me3 formation requires VACV early gene expression

**Fig 5:**
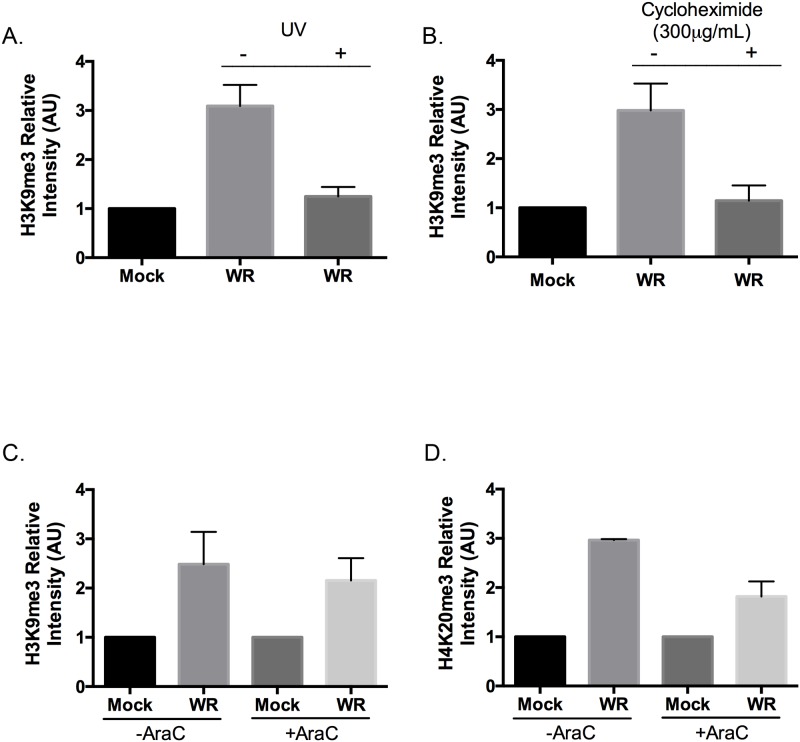
H3K9me3 and H4K20me3 formation requires VACV early gene expression. BSC-40 cells were grown on coverslips and infected for 9 hr with VACV (**A**) with or without UV inactivation, (**B**) with or without cycloheximide, or (**C, D**) with or without of AraC. The cells were fixed and stained for I3 and for H3K9me3 **(A-C)** or H4K20me3 **(D)** using specific antibodies. After imaging, the levels of nuclear H3K9me3 and H4K20me3 were quantified using FIJI and normalized relative to amounts measured in mock-infected cells. The data show the SEM of three independent experiments.

To examine whether H3K9 tri-methylation was being triggered by any of the processes relating to virion binding and entry, or delivery of virus proteins, we infected BSC-40 cells with ultraviolet (UV) light-inactivated VACV. UV-inactivated VACV can bind to cellular receptors and enter into cells; however, it cannot initiate early gene expression, which takes place prior to virion core uncoating [31, 32]. The cells were imaged 9 hr post-infection and stained to detect H3K9me3 as well as I3. I3 is a highly expressed early VACV protein and its absence showed that the UV dose was sufficient to inhibit early gene expression. In parallel we noted that H3K9me3 immunofluorescence did not significantly change following infection with UV-inactivated VACV, suggesting that viral transcription is necessary for the induction of the this response (Fig 5A).

To examine whether increased H3K9 tri-methylation was due to new protein synthesis, cycloheximide was used to inhibit protein translation [33]. The H3K9me3 levels were determined using three experimental groups of BSC-40 cells: (i) mock-treated (i.e. no drug and no virus), (ii) infected with VACV in the absence of drug, or (iii) treated with cycloheximide for 30 min prior to infection and then infected in the continued presence of the drug. The levels of I3 and H3K9me3 were then measured by immunofluorescence microscopy 9 hr post-infection. No I3 expression was detected in cells pretreated with cycloheximide, confirming the block in early viral protein synthesis. Cycloheximide treatment also blocked any alterations in the levels of H3K9me3 in VACV infected cells (Fig 5B). Thus, a productive virus infection and new protein expression are required to induce this response.

These data suggest that a virus protein, or proteins, might be required to trigger this response. As a further test, we examined what effect cytosine arabinoside (AraC) had on the formation of these chromatin marks. AraC is a cytotoxic drug that inhibits VACV DNA replication and prevents the switch to late gene expression [34, 35]. BSC-40 cells were infected (or mock-infected) with VACV and then an inhibitory concentration (80 μg/mL) of AraC was added to the culture medium 2 hr post infection. Immunofluorescence microscopy at 9 hr post-infection showed that treating infected cells with AraC did not inhibit expression of the early VACV protein I3. Moreover, exposing uninfected cells to AraC did not change the levels of H3K9me3 compared to mock-treated and uninfected cells (Fig 5C). Most critically, treating VACV-infected BSC-40 cells with AraC still permitted an increase in H3K9me3 levels compared to AraC-treated and uninfected cells. This increment was comparable to the increased amounts of H3K9me3 seen in VACV-infected cells in the absence of AraC treatment (Fig 5C). Comparable results were observed when cells were assayed for increases in the levels of H4K20me3 (Fig 5D). These results suggest that a VACV early gene, or genes, is/are responsible for the increase in H3K9me3 and H4K20me3 chromatin marks.

### Identification of the VACV gene(s) responsible for promoting histone methylation

**Fig 6:**
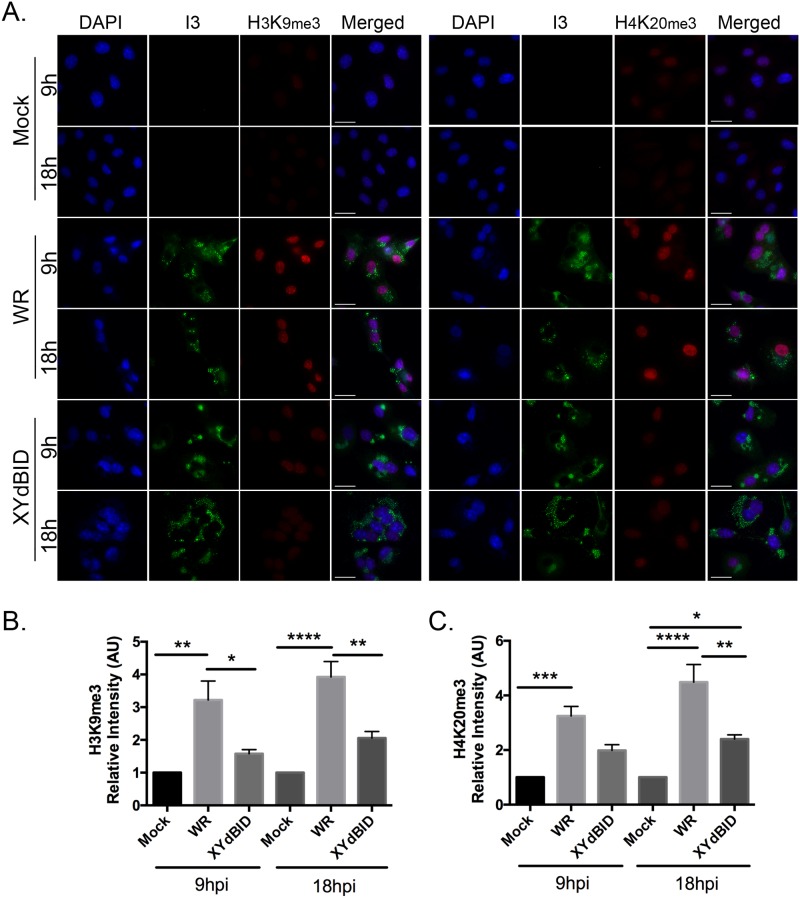
Reduced H3K9me3 and H4K20me3 formation is seen in cells infected with VACV strain XY-dBID. The strain designated as XY-dBID-VACV encodes a deletion from N1L to F4L (inclusive). BSC-40 cells were grown on coverslips and infected with VACV or XY-dBID-VACV for 9h or 18h. The cells were fixed and stained with antibodies specific to I3, H3K9me3, or H4K20me3 and for DNA (with DAPI). (**A**) Representative images showing the staining of nuclear histone markers (scale bar = 25 μm). The amounts of nuclear (**B**) H3K9me3 and (**C**) H4K20me3 were measured using FIJI and normalized relative to mock-infected cells. The data represent the SEM of three independent experiments and any statistically significant differences are noted (*P<0.05; **P<0.01; ***P<0.001; **** *P*<0.0001).

We used several approaches to narrow down and then identify the VACV gene that was promoting these reactions. First, the cycloheximide and AraC inhibitor studies suggested that an early viral gene might be responsible although this was not especially helpful since 118 of ~200 VACV genes appear to be early genes [36]. We also assumed that homologs of the gene(s) responsible would likely be absent in Leporipoxviruses. To identify the gene(s) responsible, we examined the different VACV in our collection to see whether any particular mutant was unable to modulate the levels of H3K9me3 in BSC-40 cells. We immediately noted that this phenotype characterized a virus referred to as XY-dBID-VACV. This is mutant derived from VACV strain WR and encodes an 11 kbp targeted deletion spanning genes N1L to F4L [37]. The results of these experiments are shown in Fig 6. Whereas the wild-type VACV parent again caused a 3- to 4-fold increase in the levels of H3K9me3 and H4K20me3, XY-dBID-VACV caused only a small (and not significant) change in the levels of either mark compared to non-infected cells (Fig 6B and 6C). Because XY-dBID-VACV replicates slightly slower than the parent strain (due to partial deletion of F4L encoding the ribonucleotide reductase [38]) we also examined the effects on cells at the 18 hr time point. Although there may be a gradual increase in the levels of both marks in all of the infected cells (suggesting the possibility of a second gene or some process linked to a non-specific infection-related signal), the deletion strain continued to lag significantly behind the parent strain with respect to the levels of H3K9me3 and H4K20me3. We concluded that a gene at least partially responsible for this phenotype must lie somewhere within the 11 kb region deleted in XY-dBID-VACV.

**Fig 7:**
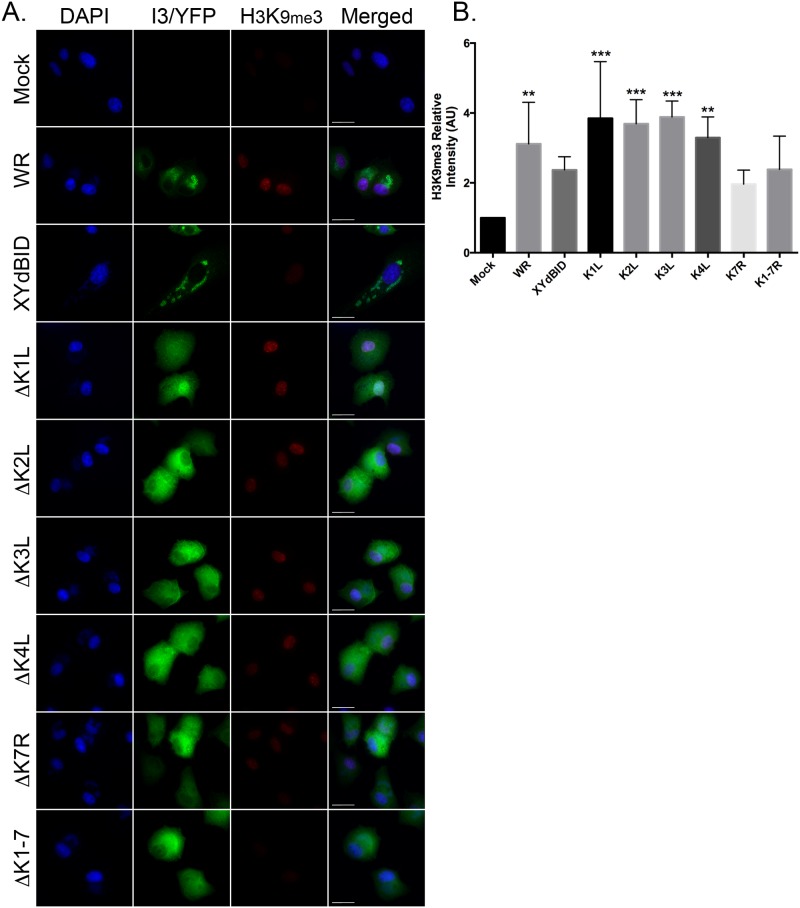
VACV ΔK7R-mutants promote less H3K9me3 and H4K20me3 formation. BSC-40 cells were grown on glass coverslips and infected (or mock infected) at a MOI of 5.0 with VACV, XY-dBID-VACV, or VACV bearing deletions in the indicated *Hin*dIII K-fragment genes. The cells were fixed and processed at 9 hr post-infection as described above. Representative microscopy images are shown (*scale* bar = 25 μm). The intensities of the nuclear H3K9me3 were quantified using FIJI and normalized to mock-infected cells. Data represent the SEM of three independent experiments. Any statistically significant differences, relative to mock-infected cells, are noted (**P<0.01; ***P<0.001).

In order to determine which gene was responsible for VACV-induced alterations in histone methylation, we screened a number of single- and multi-gene deletion mutants. In XY-dBID-VACV the deletion encompasses (in gene order) N1L, N2L, M1L, M2L, K1L, K2L, K3L, K4L, K7R, F1L, F2L, F3L and F4L. Single gene deletions were introduced into each of these genes and we also constructed two large deletion mutants spanning N1L-to-M2L and K1L-to-K7R. We infected BSC-40 cells for 9 hr with the deletion viruses and then compared the levels of H3K9me3 staining to that seen in cells infected with the wild type parent and XY-dBID-VACV. Most of the deletion mutants continued to cause a significant increase in H3K9me3 levels relative to the mock-infected cells (Fig 7). However, the levels of H3K9me3 were not increased significantly, relative to mock-infected cells, when using any of our viruses encoding mutations encompassing the K7R locus: XY-dBID-VACV, ΔK1L-K7R, and ΔK7R (Fig 7). All three mutants still caused a small (albeit not statistically significant) change in H3K9me3 levels relative to uninfected cells, again suggestive of there being more than one viral inducer of histone methylation. However, the reduction seen in all of the ΔK7R mutants suggested that K7R encoded the protein primarily responsible for the phenotype.

**Fig 8:**
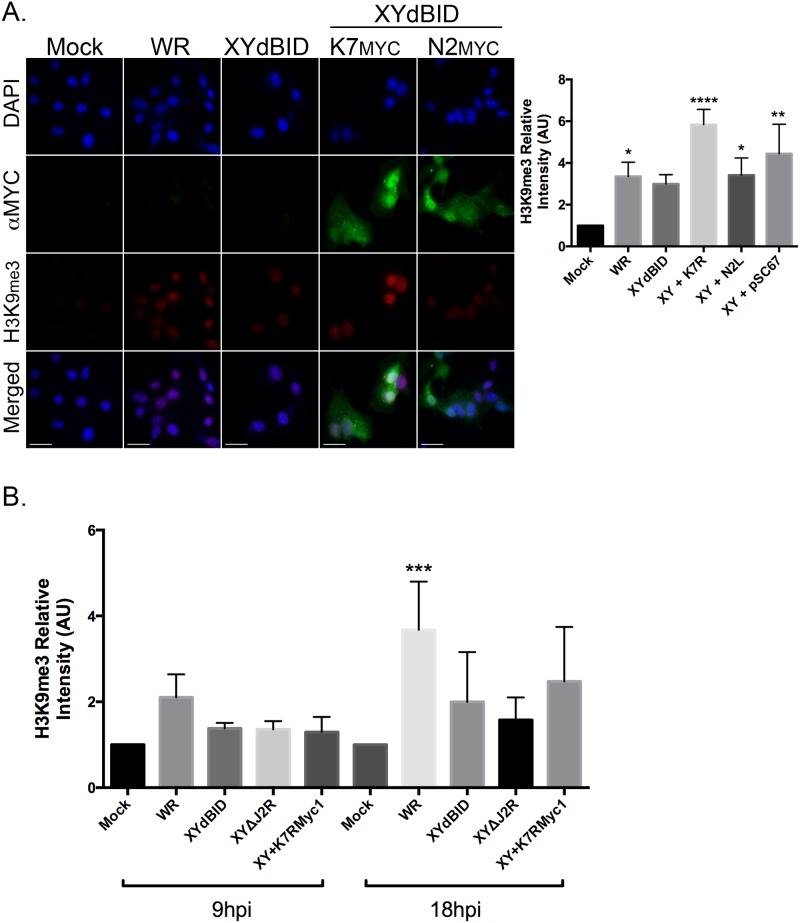
Exogenous expression of K7 and rescue of K7R in XY-dBID-VACV partially restores H3K9me formation during virus infection. **(A)** BSC-40 cells were grown on glass coverslips and infected with VACV WR or XY-dBID-VACV for 2h prior to transfection with plasmids expressing either K7-myc or N2-myc-tagged proteins. The samples were fixed 18h post infection and stained with antibodies to myc and nuclear H3K9me3. Representative images are shown (scale bar = 25 μm). (**B**) The levels of nuclear H3K9me3 were imaged as shown in panel (A), quantified using FIJI, and normalized to levels seen in mock-infected cells. Data represent the SEM of three independent experiments and statistically significant differences are noted (**P<0.01; **P<0.01, ****P<0.0001). **(C)** BSC-40 cells were infected with VACV WR, XY-dBID-VACV, XY-dBID-VACVΔJ2R, or XY-dBID-VACVK7Rmyc1 viruses, the last encoding K7Rmyc inserted into the J2R locus. The cells were fixed and stained with myc and H3K9me3 specific antibodies and counterstained with DAPI to visualize nuclei. Nuclear H3K9me3 levels were quantified using FIJI and normalized to that seen in mock-infected cells. The data show the SEM of three independent experiments and statistically significant differences are noted (*P <0.05; **P <0.01; ***P<0.001; **** *P*<0.0001).

To further test if K7R is one the genes responsible for promoting histone methylation, we examined whether restoring the locus would restore the phenotype. We anticipated that this would work based upon preliminary transfection studies. We transfected a plasmid encoding a myc-tagged version of K7 (K7R-myc) into cells infected by XY-dBID-VACV, and saw that it greatly enhanced formation of higher levels of H3K9me3 than was seen in infected cells transfected with either the empty control vector or a plasmid encoding a myc-tagged version of N2 (Fig 8A). We subsequently rescued the K7R-myc gene into the J2R locus of XY-dBID-VACV using vector pSC67-gpt/YFP and selecting for drug resistance and fluorescent plaques. A control virus was also assembled at the same time encoding the same insertion of the gpt/YFP cassette into J2R but lacking K7R. These viruses were then used to infect BSC-40 cells, and inspected using immunofluorescence microscopy. However, although some increase in H3K9me3 modification was detected in cells infected with a VACV bearing the recombined K7R-myc locus, the effect was limited and didn’t approach the significantly higher levels detected in cells infected with wild-type virus (Fig 8B). We concluded that K7 promotes some amount of heterochromatin formation in cells following VACV infection, but that it is probably not the only virus gene product encoded within the N1L-to-K7R region that does so.

## Discussion

In this study, we have shown that two types of *Orthopoxviruses* (vaccinia and cowpox viruses) induce the formation of histone marks characteristic of repressive chromatin in infected cells. These marks, H3K9me3 and H4K20me3, increased in abundance over the latter part of the infection cycle and reached a maximum 9–12 hr post-infection. However this effect was not caused by all of the viruses we tested. Infecting BSC-40 cells with either of two Leporipoxviruses (myxoma and Shope fibroma viruses), did not alter the levels of these histone marks and suggests that the effect is not a common feature of poxvirus infections in general. These studies are complicated by the fact that the absolute increase in signal is modest, up to 4-fold by immunofluorescence microscopy and perhaps only 2-fold by flow cytometry. This necessitates using several independent experiments and many replicates in order to detect small experimental signals buried in a noisy global background. However, it is likely that the effects at the gene level could be much more profound, given that these kinds of chromatin marks are typically non-randomly located at specific sites. If one were to pursue these studies further (especially the mutational screening) it would be very helpful to identify one or more of these sites and use site-specific methylation as a more robust experimental readout than the optical methods we have been limited to using.

A number of viral pathogens have been reported to perturb chromatin structure through altering histone methylation states (reviewed in [39]). Perhaps most closely studied has been the manner in which herpes simplex virus regulates latency, in part, by depositing facultative heterochromatin on the viral DNA. African swine fever virus causes a more general effect, similar to what we see here, where infection causes the widespread appearance of H3K9me3 marks indicative of heterochromatinization of the host cell genome [40]. Very recent studies have also highlighted a still incompletely understood process in which influenza infection causes type-I interferon-induced upregulation of the lysine methyltransferase Setdb2, and the subsequent silencing of antiviral effectors in macrophage by H3K9me3 formation [41, 42]. In this regard it is striking that we saw high levels of H3K9me3 marks formed in VACV-infected HEL cells along with extensive associated cytopathic effects (S1 Fig), but whether one phenotype (heterochromatin formation) is causally related to the other (enhanced virus-mediated cytotoxicity) remains to be demonstrated. There is also an extensive literature showing that bacterial lipopolysaccharide (LPS) can tolerize cells to LPS through a process involving the formation of facultative heterochromatin on NFκB-regulated promoters. (This process is discussed in greater detail below.) The reasons for why host cells would become unresponsive to toxicants like LPS are still imperfectly understood, but may be related to the importance of limiting the damage that can be caused by acute inflammatory immune responses. If not regulated, severe systemic inflammation can lead to organ failure and consequently gene silencing is detected in cells that have been exposed to these conditions. However, this immune suppression creates a risk of secondary infections, such as the bacterial infections that are often seen following influenza infection.

Our deletion and complementation analysis leads us to conclude that the effects we see are partly mediated by the VACV K7R gene. Bioinformatics analysis (http://athena.bioc.uvic.ca/virology-ca-tools/vocs/) finds K7R homologs encoded only by viruses in the *Orthopoxvirus* genus (variola, monkeypox, horsepox, camelpox, taterapox, and raccoonpox viruses). Oddly, the gene appears to be mutated and inactivated in all of the ectromelia (mousepox) virus strains sequenced to date. There are no obvious K7R homologs encoded by either myxoma or Shope fibroma viruses or indeed any other type of poxvirus. The VACV K7R gene product has been studied previously both structurally and from an immunological perspective. The K7 protein has been crystallized and adopts a monomeric structure resembling a Bcl-2 fold [43, 44]. It is one of many VACV proteins that have been shown, or are predicted, to exhibit this common structural feature [45–47]. K7 interacts with TRAF6, IRAK2, and the DEAD-box RNA helicase DDX3 and can inhibit both TLR-dependent NF-κB activation as well as inhibiting IRF-dependent activation of the IFN-β promoter [43, 48]. As a consequence, deleting K7R reduces VACV virulence in murine models and also alters the nature of the immune response to infection [49].

TRAF6 and IRAK2 are components in the signalling cascade leading from TLR4 to NF-κB activation and the production of pro-inflammatory cytokines [47]. DDX3 is a key component in the signalling cascade that leads to induction of genes encoding Type I interferons. How K7 might function to perturb these pathways is not well established, although the simplest model would involve K7 binding to DDX3, TRAF6 and/or IRAK2, and simply interfering with downstream signalling. What is unclear is how K7 might then promote histone methylation, and whether this activity is related in any way to the known activities of the protein. Certainly nothing about the K7 sequence would suggest that it is a lysine methyltransferase. Moreover, our preliminary studies showed that H3K9 tri-methylation is not observed in VACV-infected mouse embryonic fibroblasts lacking the SUV39H1 and SUV39H2 lysine methyltransferases suggesting that this process is ultimately catalyzed by cellular systems.

Although the links remain to be established, what we see here bears a resemblance to the phenomenon of LPS tolerance. Under conditions where cells have been LPS tolerized, the promoters of pro-inflammatory genes like IL-1β are silenced by facultative heterochromatin and this seems to be formed through the actions of histone methyltransferases recruited to a labile complex composed of IκBα RelB and HP1 (heterochromatin protein 1) [50, 51]. It is noteworthy that when these pathways are activated by IRAK2- and TRAF6-mediated signalling, it causes the phosphorylation and degradation of IκBα and permits NF-κB recruitment to target promoters [47]. The corollary might be that IκBα would be stabilized in VACV-infected cells, where K7 is expected to inhibit signalling through IRAK2 and TRAF6, and this could favour formation of gene silencing complexes. It is also noteworthy that RelB is one of a small number of host genes upregulated in VACV-infected cells [12] as it is in LPS-treated cells [52]. This has to be an oversimplification of the situation, especially because N1 might also be expected to inhibit these reactions and we could not detect a significant decrease in histone methylation in cells infected with viruses bearing a deletion spanning the N1L-M2L region. Moreover, these models have been developed in the context of LPS stimulation in cell types unrelated to BSC-40 cells. Nevertheless the precedent exists for suppressing proinflammatory gene expression through gene silencing. A key prediction of this model is that these virus-induced histone marks should be located in a specific class of promoters (for example the IL-1β locus) and this can be tested in future using techniques like chromatin immunoprecipitation assays.

## Materials and methods

### Cells and viruses

African green monkey cells (BSC-40) were purchased from ATCC and grown in minimum essential medium (MEM) with 1% L-glutamine, 1% non-essential amino acids, 1% sodium pyruvate and 1% antibiotic/antimycotic plus 5% fetal bovine serum. Primary human embryonic lung (HEL) fibroblasts were obtained from Dr. James Smiley at the University of Alberta and grown in Dulbecco’s MEM with 1% L-glutamine, 1% antibiotic/anitimycotic plus 10% fetal bovine serum. These cells all tested negative for mycoplasma by PCR (Invitrogen). Early passage cells were preferred for use in these experiments as the H3K9me3 and H4K20me3 background increases with extended passage. VACV (strains WR and Copenhagen), cowpox virus (strain Brighton Red), and Shope fibroma virus were also originally purchased from ATCC. Myxoma virus (MYX-LacZ, strain Lausanne) was a gift from Dr. Grant McFadden (University of Florida) [53]. XY-dBID-VACV was constructed previously as described [37].

### Plasmids and recombinant viruses

A number of VACV mutant strains were generated for these studies encoding either single (ΔM1L, ΔM2L, ΔK1L, ΔK2L, ΔK3L, ΔK4L, ΔK7R, ΔF1L and ΔF2L) or more extended (ΔN1L-M2L and ΔK1L-K7R) gene deletions. To delete the VACV genes, we used a plasmid bearing a copy of an EcoGPT marker fused to yellow fluorescent protein (pDGloxPKO^DEL^ [54]). DNAs encoding sequences flanking the desired gene targets were cloned flanking the selection marker, sometimes leaving behind a small portion of the C-terminus of the targeted genes to avoid disrupting adjacent promoters. BSC-40 cells were infected with VACV strain WR at MOI = 2 and then transfected, 2 hr later, with 2 μg of linearized plasmid using Lipofectamine 2000 (Invitrogen). The progeny were harvested next day and the recombinant viruses were isolated using two rounds of drug selection [55] in liquid culture, followed by three rounds of plaque purification under agar. To restore the K7R gene, the PCR and two primers (5’-GTCGACATGGCGACTAAATTAGAT-3’ and 5’-GCGGCCGCTCACAGATCTTCTTCAGAAATGAGTTTTTGTTCATTCAATTTTTTTTCTAGA-3’) was first used to add sequences encoding a myc tag to the C-terminus of the K7R gene, and the product was then cloned into pSC66-gpt/YFP (a gpt/YFP cassette replaced the LacZ gene in pSC66 [56]). The plasmid was then transfected into cells previously infected with XY-dBID-VACV and the recombinant viruses (encoding K7R regulated by its native promoter and inserted into the J2R locus) were selected using mycophenolic acid and purified as described above. The PCR was used to confirm the identity of all the viruses.

### Antibodies and other reagents

The antibodies used in these studies were mostly purchased from commercial sources. These included antibodies recognizing a Myc-tag (Cell Signalling), H3K9me3 (Active Motif), and H4K20me3 (Abcam). The VACV I3L 10D11 monoclonal antibody was from laboratory stocks. The Alexa-fluor 488 and Cy-5 conjugated secondary antibodies were purchased from Molecular Probes.

### Microscopy and image analysis

Immunofluorescence microscopy was used for quantitative analysis of chromatin marks. BSC-40 cells were grown on glass coverslips, infected with virus at MOI = 5, cultured as indicated, and then fixed on ice for 30 min in phosphate buffered saline (PBS) containing 4% paraformaldehyde. The fixed cells were then treated with 0.1 M glycine followed by blocking buffer [PBS supplemented with 3% bovine serum albumin and 0.1% Triton X-100 (MP Biochemicals)]. The cells were then stained overnight in 1:1000 diluted primary antibody in blocking buffer, and then counterstained for 1–3 hr in 1:2000 diluted secondary antibody. The cells were stained with 5 ng/ml DAPI (Sigma), washed in PBS supplemented with 0.1% Triton X-100, and mounted in mounting media [0.1mg/mL Mowoil (Molecular Probes), 0.1M Tris·HCl (pH 6.8), 25% w/v glycerol]. The samples were imaged using an Applied Precision Deltavision microscope at 60× magnification (N.A. = 1.42) and processed using SoftWorx (v4.1.2). Image analysis was performed using ImageJ or FIJI [57]. All images were collected under conditions that avoided signal saturation, and the same gain settings, background corrections, and a linear gamma factor were used across all samples in a given experiment. The relative fold change of the intensities was calculated by dividing the normalized intensities of the experimental group (e.g. virus-infected cells) to that of the control group (mock-infected cells). Unless otherwise indicated, all experiments were performed using three independent replicates. We report the average (mean) fold change and standard deviations. GraphPad was used to determine statistical significance using a one-way ANOVA.

### Flow cytometry

BCS40 cells were infected (or mock-infected) at MOI = 5 for 9 hr and then detached with TrypLE Express solution. The cells were recovered by centrifugation for 5 min at 300×g, and washed twice with PBS. The cells were then stained with a fixable viability dye eFluor 780 (1:1000) for 30 min on ice, centrifuged and fixed with IC fixation buffer (eBioscience) for 30 min at room temperature, and washed twice with FACS Buffer (PBS containing 5mM EDTA and 1% FCS). The cells were treated for 15 min at room temperature with 1× permeabilization buffer (eBioscience) containing 0.1% Triton-X and recovered by centrifugation at 400×g for 7 min, stained for 3.5 hours at room temperature with antibodies directed against I3 (diluted 1:10,000) and H3K9me3 or H4K20me3 (diluted 1:1000), and washed twice with permeabilization buffer. After washing with PBS, the cells were incubated for 45 min at room temperature with Cy5 and AF488 secondary antibodies (both diluted 1:1000). Finally, the cells were washed twice with PBS and analyzed using FlowJo software and a FACS-LSR Fortessa cell analyzer (BD Biosciences). Briefly, we gated on the live single cell population and measured the median fluorescence intensity of either H3K9me3 or H4K20me3. Judging by the I3 levels approximately 85–95% of the cells were infected at 3hr and 98–99% by 6hr post-infection.

## Supporting information

S1 Fig VACV infection, but not SFV infection, increases the level of H3K9me3 in primary human fibroblast cells. HEL fibroblasts were grown on coverslips and subsequently infected with VACV WR or SFV. At 9h and 24h post infection the cells were fixed and stained to detect VACV I3 and (**A**) H3K9me3. DNA was counterstained with DAPI. The presence of viral factories (stained with DAPI) was used to confirm infection with SFV, as the I3 antibody does not cross-react in Leporipoxviruses. Representative images are shown (scale bar = 25 μm). Nuclear (**B**) H3K9me3 and signal intensities were quantified using FIJI imaging analysis software and normalized to mock-infected cells. We show the SEM of three independent experiments. Statistically significant differences are noted (*P<0.05; **** *P*<0.0001).
(TIF)
