# Supplementary figures and images for: The vaccinia virus K7 protein promotes histone methylation associated with heterochromatin formation
Source: PLoS One. 2017 Mar 3;12(3):e0173056. doi: 10.1371/journal.pone.0173056 (PMC5336242; doi:10.1371/journal.pone.0173056)

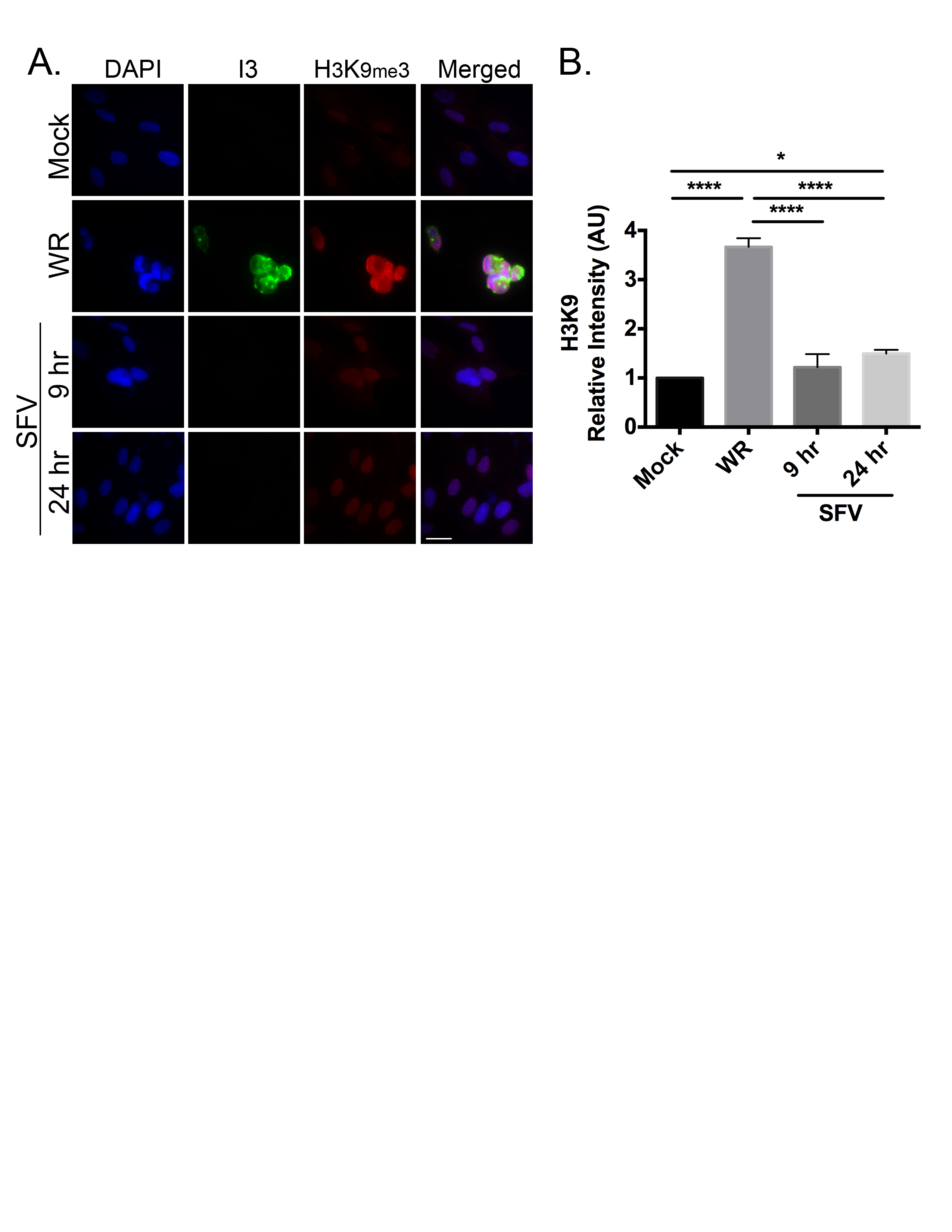

Supplement: S1 Fig — HEL fibroblasts were grown on coverslips and subsequently infected with VACV WR or SFV. At 9h and 24h post infection the cells were fixed and stained to detect VACV I3 and (A) H3K9me3. DNA was counterstained with DAPI. The presence of viral factories (stained with DAPI) was used to confirm infection with SFV, as the I3 antibody does not cross-react in Leporipoxviruses. Representative images are shown (scale bar = 25 μm). Nuclear (B) H3K9me3 and signal intensities were quantified using FIJI imaging analysis software and normalized to mock-infected cells. We show the SEM of three independent experiments. Statistically significant differences are noted (*P<0.05; **** P<0.0001). (TIF) [file pone.0173056.s001.tif]
